# EU-Approved Rapid Tests for Bovine Spongiform Encephalopathy Detect Atypical Forms: A Study for Their Sensitivities

**DOI:** 10.1371/journal.pone.0043133

**Published:** 2012-09-11

**Authors:** Daniela Meloni, Aart Davidse, Jan P. M. Langeveld, Katia Varello, Cristina Casalone, Cristiano Corona, Anne Balkema-Buschmann, Martin H. Groschup, Francesco Ingravalle, Elena Bozzetta

**Affiliations:** 1 Centro di Referenza Nazionale per le Encefalopatie Animali, Istituto Zooprofilattico Sperimentale del Piemonte, Liguria e Valle d'Aosta, Turin, Italy; 2 Central Veterinary Institute of Wageningen UR, Lelystad, The Netherlands; 3 Friedrich-Loeffler Institut, Federal Research Institute for Animal Health, Insel Riems, Germany; The Scripps Research Institute Scripps Florida, United States of America

## Abstract

Since 2004 it become clear that atypical *bovine spongiform encephalopthies* (BSEs) exist in cattle. Whenever their detection has relied on active surveillance plans implemented in Europe since 2001 by rapid tests, the overall and inter-laboratory performance of these diagnostic systems in the detection of the atypical strains has not been studied thoroughly to date. To fill this gap, the present study reports on the analytical sensitivity of the EU-approved rapid tests for atypical L- and H-type and classical BSE in parallel. Each test was challenged with two dilution series, one created from a positive pool of the three BSE forms according to the EURL standard method of homogenate preparation (50% w/v) and the other as per the test kit manufacturer's instructions. Multilevel logistic models and simple logistic models with the rapid test as the only covariate were fitted for each BSE form analyzed as directed by the test manufacturer's dilution protocol. The same schemes, but excluding the BSE type, were then applied to compare test performance under the manufacturer's versus the water protocol. The *IDEXX HerdChek ® BSE-scrapie short protocol* test showed the highest sensitivity for all BSE forms. The *IDEXX® HerdChek BSE-scrapie ultra short protocol*, the *Prionics® - Check WESTERN* and the *AJ Roboscreen® BetaPrion* tests showed similar sensitivities, followed by the *Roche® PrionScreen*, the *Bio-Rad® TeSeE™ SAP* and the *Prionics® - Check PrioSTRIP* in descending order of analytical sensitivity. Despite these differences, the limit of detection of all seven rapid tests against the different classes of material set within a 2 log_10_ range of the best-performing test, thus meeting the European Food Safety Authority requirement for BSE surveillance purposes. These findings indicate that not many atypical cases would have been missed surveillance since 2001 which is important for further epidemiological interpretations of the sporadic character of atypical forms.

## Introduction

*Transmissible spongiform encephalopathies* (TSEs) or prion diseases include a group of progressive, neurodegenerative as yet untreatable disorders affecting several mammalian species, including Creutzfeldt-Jakob disease (CJD) in humans, bovine spongiform encephalopathy (BSE) in cattle and scrapie in small ruminants. TSEs are characterized by the concentration of an anomalous isoform (PrP^Res^) of the natural prion protein (PrP^c^) in the central nervous system (CNS) and peripheral tissues. PrP^res^ differs from PrP^c^ in its aggregated state and partial protease resistance. These characteristics are exploited by the majority of the methods currently used for TSE diagnosis. The protease resistant disease related PrP entity, varies in its extent of degradation by proteinase K (PK) which is influenced by the strain-dependent conformational variations of the secondary and tertiary structure of PrP^res^. In different TSE strains, the pathological prion protein displays disease-specific features such as different cleavage sites after proteolytic treatment, glycosylation profile, and deposition patterns, which make strain identification possible [1].

The existence of different BSE strains was discovered in 2004. The classical form (C-BSE) coexists with the atypical H-type BSE (H-BSE) originally described in France [2] and the L-type BSE (L-BSE), also known as bovine amyloidotic spongiform encephalopathy (BASE), an unusual form of BSE first identified in Italy [3]. Their diagnostic differentiation is based mainly on the molecular features of the PrP^res^ identified by Western blot analysis. After PK digestion, PrP^res^ shows a triplet of non-, mono-, and diglycoforms, which, while expressing their quantitative ratio and migration positions, peculiarly typify the original BSE strain [4]. H- and L-BSE have a higher or a lower discernible molecular mass of unglycosylated PrP^res^, respectively, at Western blot analysis; in addition, L- BSE has smaller proportion of diglycosylated PrP^res^ with levels between 40–55% than C-BSE where values range between 60–80%. Subsequently, occurrence of the two atypical forms to several European countries, Japan and North America has been reported. The origin of different BSE forms is still cryptic. C-BSE, which was isolated during the epizootic disease, was postulated to have occurred after the recycling of a scrapie agent insufficiently deactivated in destructor plants [5], [6]. However, this has been questioned after the detection of atypical BSE forms that seem to occur spontaneously in older cattle [7] and that, under certain circumstances, are able to change their biochemical properties into those of classical BSE. The accidental use of bovine material derived from an animal that had succumbed to a spontaneous form of BSE in the feed and food production may therefore also have been the origin of the BSE crisis. Such hypothesis seems to be also compatible with the peculiar distribution by year of birth of cattle affected by atypical BSEs, in comparison to bovines affected by C-type BSE observed in France [8]. Moreover, it has been shown in several transmission experiments to primates [9], [10] and in human and bovine PrP transgenic mice [11], [12] that L-type BSE seems to have a higher zoonotic potential than C-type BSE. The use of rapid tests that are able to reliably detect such cases is therefore crucial in the frame of the protection of the consumer from an accidental exposure to the BSE agent.

Until 1999 EU surveillance systems for bovine spongiform encephalopathy (BSE) were primarily passive, *i.e*. relying on the examination of diseased adult cattle showing clinical signs reported to the veterinary authorities, in compliance with the Decision 98/272/EC which modifies Decision 94/474/EC [13]. Brains were examined by histopathology and immunhistochemistry for PrP^res^ identification. Rapid molecular diagnostic assays became officially available in the late 1990s. With the enforcement of Regulation (EC) No. 999/2001 [14] the use of rapid tests became mandatory: a large number of countries subsequently detected the first BSE cases.

To provide dependable tools for an active surveillance system, in 1999 the European Commission (EC) carried out the first scientific evaluation of four new rapid *post mortem* BSE tests to assess their diagnostic accuracy and analytical sensitivity on brain tissue from clinically affected bovines [15]. Subsequent EU validation exercises enhanced the estimating parameters, including test robustness on autolyzed samples and testing of negative field samples to address the test specificity and to simulate routine activity [16], [17], [18].

To date, the EC has assessed 19 rapid tests in the frame of three “successive” evaluations and approved 9 for survey purposes [19].

In 2009 the Community Reference Laboratory (EURL) for TSEs assessed the analytical sensitivity of all the currently approved TSE rapid tests to determine their continued suitability for active surveillance plans [20]. The analytical sensitivity study was then evaluated by the European Food Safety Authority (EFSA) [21], [22] on the basis of current EFSA requirements for the evaluation of TSE rapid *post mortem* tests [23].

In that context, the lowest limit of detection (LOD) of rapid tests approved for the diagnosis of TSEs in bovines was assessed. The pre-prepared positive and negative dilution series (EURL protocol) were compared with the manufacturer's dilution series. The rapid tests with a LOD poorer than 2 log_10_ as compared to the best-performing assay could not be recommended for use in the frame of BSE monitoring in cattle and TSE in small ruminants within the EU.

At same time, the BIOHAZ Panel recommended that a similar study should have been conducted with regard to the other TSE strains. Furthermore, because experimental transmission of atypical BSE prions suggests that they might be more insidious than classical BSE [24], the assessment of approved rapid-test performance on detecting atypical BSE strains remains a priority.

The aim of this study was to compare the analytical sensitivity of all presently EU-approved rapid *post mortem* tests for the detection of atypical BSE forms in bovines by assessing their lower LOD against atypical L- and H-type BSE. The outcome will be of interest for the interpretation of epidemiological surveillance data of all three BSE types.

## Materials and Methods

### Study Design

Consistent with the methodology of the EFSA analytical sensitivity study, the strategy of the Italian TSE National Reference Laboratory (NRL) was to compare the performance of all approved rapid tests against the same sample pools. Thus, the test results could be directly compared and their performance ranked according to their respective LOD.

Each test was challenged with decreasing amounts of confirmed BSE-positive material in a consistent background of negative material prepared following two protocols: the one using the EURL standard method of homogenate preparation (50% w/v protocol) [23] and the other as directed by the test kit manufacturer's instructions. This was done to permit comparison between the two preparation protocols.

The study design was set up to account for several confounders (e.g., the operator, the day of the test, plates, etc.). Factors were controlled by minimizing variability (e.g., all tests were performed by only two operators) and fitting multilevel logistic models in which the confounders were set as random or fixed effects [25], [26], [27].

The first step was a basic descriptive analysis. The second involved only the manufacturer's protocol, and a multilevel logistic model was fitted for each BSE type in order to compare test kit performance. The date of testing execution constituted the first level of the models (level 1); replicated crossover and nesting of the plates were the second level (level 2). The intraclass correlation coefficient (ICC) of the replicates obtained from these models allowed us to verify that their residual variance was due only to the replicates [25], [26], [27]. In this case, when the number of positive replicates was monotone decreasing in the dilutions, we could neglect the fixed effect of the dilution and focus instead on the effect of the different test kits. In the third step, simple logistic models [28], [29] were fitted with the specific test kit as the only covariate.

Finally, the test results obtained under the two preparation protocols were compared. As mentioned, we fitted the multilevel logistic models not referring to each BSE type but instead to each test kit. Seven simple logistic models [28], [29] one for each test, were then fitted: the interaction between BSE type and protocol was the only covariate in the model.

### Tissue Background

Briefly, atypical L-BSE tissue was obtained from two Italian field cases. The atypical H-BSE tissue pool was provided to the Italian NRL by the Friedrich-Loeffler-Institut (FLI) (Germany) and originated from German calves experimentally inoculated intracranially [24]. Two C-BSE pools were included in the study, one strongly reacting at confirmatory Western blot, the second weakly. This was done in order to have reference data on known matrices for the comparison of unexplored results with atypical BSE. The strong C-BSE type tissue included a pool of five Dutch regularly slaughtered field cases (collected and tested in the frame of statutory BSE surveillance plane) provided by Central Veterinary Institute of Wageningen UR (CVI). The weak C-BSE tissue was a mixture of two Italian natural cases. All positive tissues originated from brain stem area and were confirmed by discriminatory Western blot analysis [4]. The negative tissue was created from 30 bovine brainstems randomly selected from Italian slaughtered surveillance samples which had tested negative at the *IDEXX® HerdCheck BSE-scrapie ultra short protocol* test [30] and confirmatory Western blot.

All details pertaining to sample origin were recorded.

### Preparation of Diagnostic Test Material

To ensure that the samples would be homogeneous, they were prepared using the Veterinary Laboratory Agency (VLA) standard methods for TSE QA sample production (Veterinary Laboratory Agency, Standard Operating Procedure, “Instruction for the homogenisation and dilution of brainstem for preparation of QA Samples” – personal communication).

Accordingly, four CNS tissue pools (L, H, C strong, and C weak) were prepared from L-BSE-positive, H-BSE-positive, C-BSE strong and C-BSE weak positive tissues, respectively. The 100% CNS tissues were trimmed, pooled, mildly minced with scalpels, and then treated with a low-speed hand-held homogenizing unit for 30 s. A negative pool was prepared as described above. Each BSE-positive macerate pool was diluted in pre-homogenized negative tissue to obtain 2 base logarithm dilutions series down to 1∶1024. As the 1∶1024 dilution of the C-BSE strong pool tested positive at the *IDEXX® HerdCheck BSE-scrapie short protocol*, further 1∶2048 and 1∶4096 dilutions of the same tissue were investigated, with negative results.

To set up the dilution series, one half of the BSE-positive pools was prepared under the EURL homogenization protocol in *nuclease*-free water (50% w/v) using a low-speed hand-held homogenizing unit for a total of 90 s in three successive treatments. Each dilution underwent a final homogenization cycle to ensure the preparation was mixed thoroughly. All the homogenates were aliquoted into test-specific pre-labelled grinding tubes as directed by the manufacturer's instructions and stored at −20°C.

The other half of the BSE-positive and negative starting tissue pools was distributed in the manufacturer's tissue-disruption supports for the different test kits and then immediately submitted to the specific protocols as per the manufacturer's instructions.

Each dilution was tested in triplicate by each rapid test. The test panel consisted of 150 aliquots, with 30 samples per pool, for each dilution protocol.

### Testing Exercise

The tests included in the study were those approved according to Regulation (EC) No. 999/2001 amended by Regulation 162/2009. Enfer Scientific [31] declined to participate in the study. The *Prionics® - Check LIA BSE Antigen Test Kit* [32] had been withdrawn from the market at the time the study was conducted.

A unique batch of each rapid test specifically provided for this study by the manufacturers largely before the relative expiring date was used for all the analyses. One rapid test was performed per day and all the dilution series were tested in triplicate. One out of three positive results interpreted according to the test specifications was selected as the criterion for judging the overall result as positive. For the evaluation of the *Prionics® - Check WESTERN* [33], the samples were considered positive if they exhibited a signal with a three-band pattern. A more diffuse pattern of PrP^res^ with the top band clearly visible, as reported by the manufacturer, was considered positive as well.

The laboratory test exercise was completed within 15 days from the starting point of generating and freezing the aliquots.

## Results

The analyses of the different BSE samples – C-type strong, C-type weak, H-type and L-type – under both the EURL protocol or the manufacturers' protocol indicated that in principle, all tests were able to detect the different types of BSE though at different sensitivity (Table 1).

**Table 1:** Detection limits obtained by the different rapid tests for the different BSE forms. The number of positives out of three replicates is also reported.

Test	Weak C – BSE		Strong C – BSE		L – BSE		H – BSE		Number of false positive/number of negative samples tested
	Manufacturer prepared dilutions	50% w/v	Manufacturer prepared dilutions	50% w/v	Manufacturer prepared dilutions	50% w/v	Manufacturer prepared dilutions	50% w/v	
IDEXX® HerdCheck BSE-scrapie Short	1∶64	1∶16	1∶1024	1∶512	1∶512	1∶512	1∶256	1∶512	0/30
	3/3	3/3	2/3	3/3	3/3	2/3	3/3	2/3	
IDEXX® HerdCheck BSE-scrapie Ultra Short	1∶32	1∶16	1∶512	1∶512	1∶256	1∶128	1∶128	1∶64	0/30
	3/3	3/3	3/3	3/3	3/3	3/3	1/3	3/3	
Bio-Rad ® TeSeE TM SAP	1∶2	-	1∶64	1∶4	1∶16	1∶4	1∶32	1∶32	0/30
	3/3	0/3	3/3	3/3	3/3	3/3	2/3	3/3	
Prionics®-Check Western	1∶32	1∶16	1∶128	1∶64	1∶256	1∶32	1∶128	1∶128	0/30
	1/3	3/3	3/3	2/3	1/3	3/3	3/3	1/3	
Prionics®-Check PrioSTRIP	1∶4	1∶2	1∶32	1∶16	1∶16	1∶16	1∶16	1∶16	0/30
	1/3	1/3	2/3	2/3	3/3	3/3	3/3	3/3	
AJ Roboscreen® BetaPrion	1∶32	1∶16	1∶128	1∶64	1∶512	1∶128	1∶128	1∶32	0/30
	2/3	3/3	2/3	2/3	2/3	3/3	3/3	3/3	
Roche PrionScreen®	1∶8	1∶2	1∶64	1∶16	1∶16	1∶16	1∶32	1∶64	0/30
	3/3	3/3	2/3	3/3	3/3	1/3	3/3	2/3	

The ability of the rapid tests to identify positive replicates clearly differed between the tests when increasing dilutions were compared within the manufacturers' protocol and under the EURL 50% w/v protocol. Under the manufacturer's dilution protocol, the sensitivity of the *IDEXX® HerdCheck BSE-scrapie short protocol* test for all BSE types was higher than that of the other rapid tests. The sensitivity of the *IDEXX® HerdCheck BSE-scrapie ultra short protocol*, Prionics*® - Check WESTERN*, and *AJ Roboscreen® BetaPrion* [34] was similar, followed in decreasing order by the *Roche® PrionScreen* [35] *Bio-Rad® TeSeE™ SAP* [36] and *Prionics® - Check PrioSTRIP* [37], the last two of which displayed the lowest analytical sensitivity, notably for L-BSE.

The multilevel models fitted in the second step of the statistical analysis confirmed that the residual variance was almost entirely due to the replicates. For all BSE types, the ICC of the replicates was higher than 0.99. Therefore, apart from a few exceptions, it was assumed that the three replicates for each dilution would have the same result, whereupon a simplified logistic model was adopted. The *IDEXX® HerdCheck BSE-scrapie short protocol* was taken as the reference test, as it provided the highest analytical sensitivity for all BSE forms.

The logistic models showed that a loss of sensitivity up to two dilutions lower than the best-performing test was not statistically significant. Testing with the C-BSE strong pool showed that only the *IDEXX® HerdCheck BSE-scrapie ultra short protocol* test compared favourably with the *IDEXX® HerdCheck BSE-scrapie short protocol* test; while the sensitivity of the *AJ Roboscreen® BetaPrion*, *IDEXX® HerdCheck BSE-scrapie ultra short protocol*, *Prionics® - Check WESTERN* for detecting the other BSE types was not statistically different from that of the reference test.

**Figure 1:**
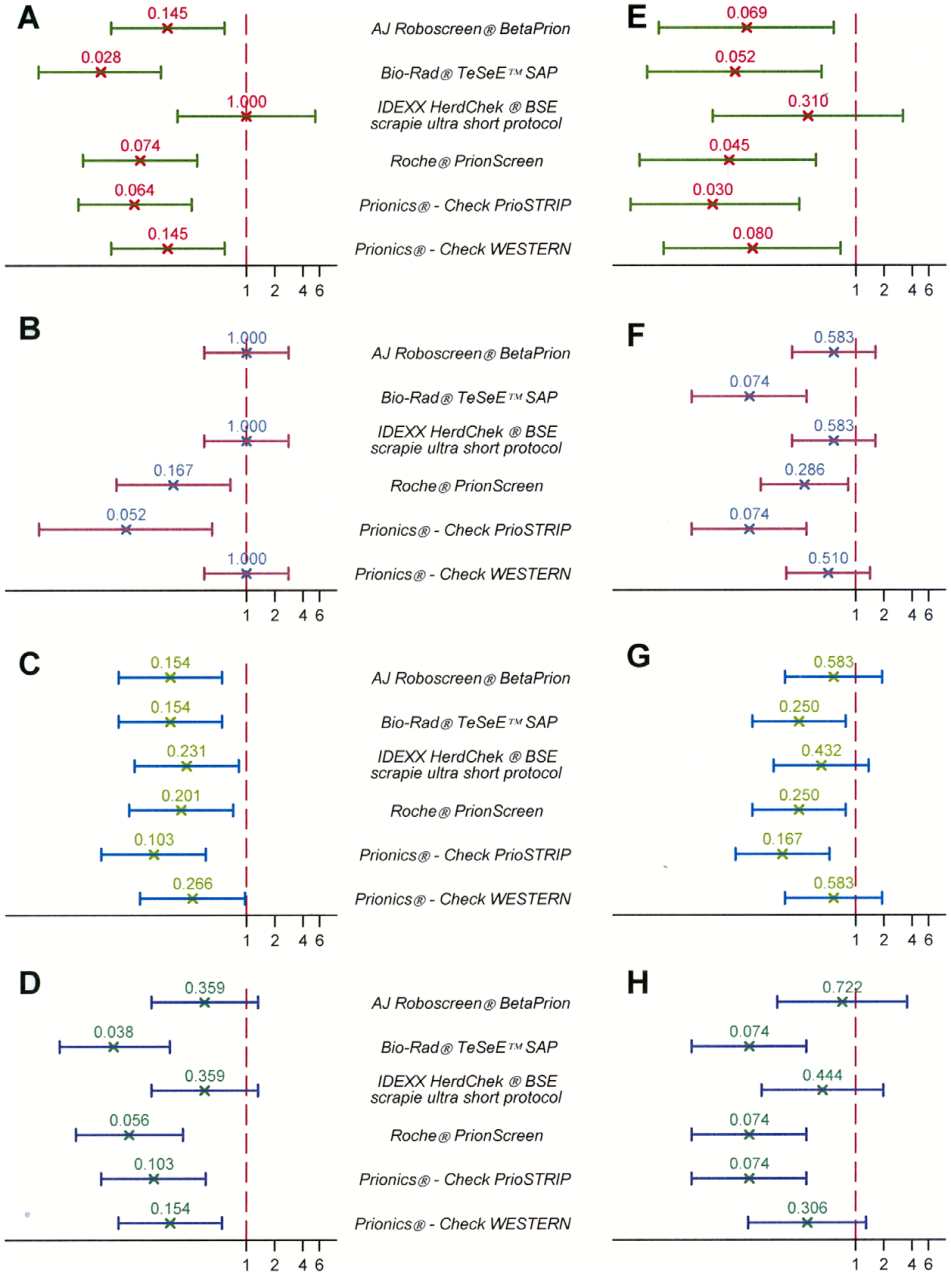
Test performance compared to the *IDEXX® HerdCheck BSE-scrapie short protocol*. The vertical axis reports the different rapid tests challenged. The horizontal axis reflects the odds ratio magnitude using *IDEXX® HerdCheck* BSE-scrapie short protocol as reference test. Panels A, B, C, D (left column) report the results obtained under the w/v protocol; panels E, F, G, H (right column) display the results under the manufacturers' instructions. BSE forms studied: panels A, E: strong C-type; panels B, F: weak C-type; panels C, G: H-type; panels D, H: L-type. All the weak C type water dilutions series tested negative with Bio-Rad® TeSeE™ SAP (notably, the optical densities were for all the three replicates of the 1∶2 dilution just under the cut-off value), thereby, the odd ratio could not be calculated (Subfigure B).

To further discriminate between the quality of performance of the different test systems, logistic models were applied on the data obtained. On the basis of the odds ratio (OR) magnitude, each test can be ranked using the *IDEXX® HerdCheck BSE-scrapie short protocol* as reference test and it can be concluded that a higher OR is related to higher sensitivity. Ranking obtained under the manufacturer's protocol did not differ from that obtained under the water protocol (Figure 1).

Comparison of test performance under the two dilution protocols based on the descriptive analysis (Table 1) showed that all seven tests had a higher analytical sensitivity for the BSE-positive samples prepared under the manufacturer's protocol than those prepared as *per* the water protocol for all BSE types, except for H-BSE, toward which the tests performed generally better with the 50% w/v homogenates.

**Figure 2:**
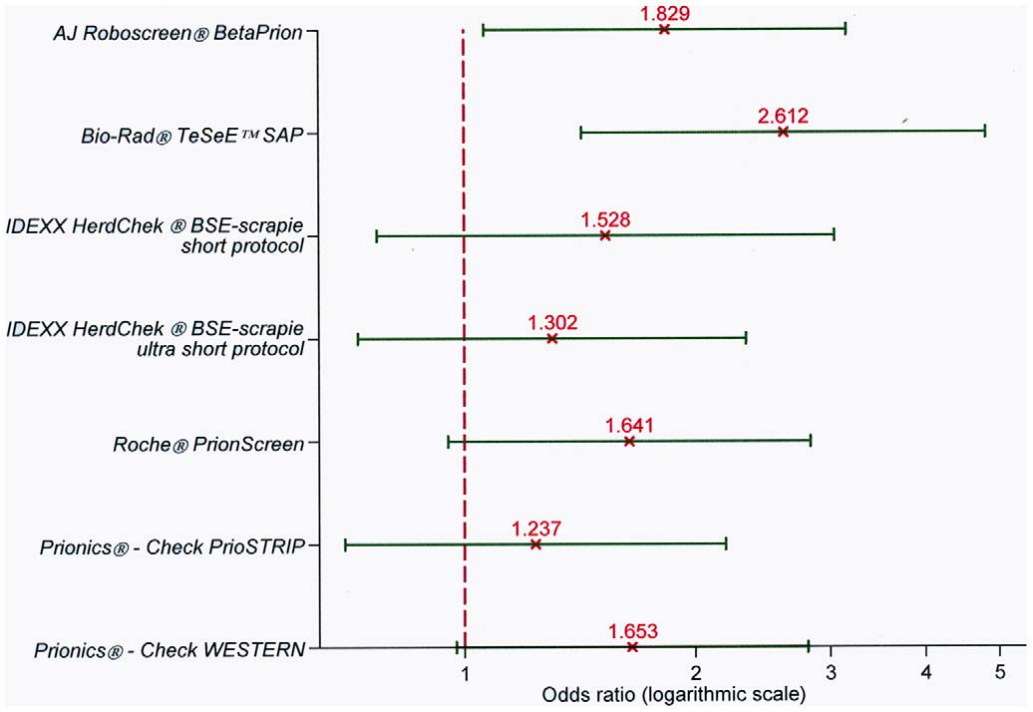
Comparison of test performance under the manufacturer's dilution protocol *versus* the 50% w/v protocol. The vertical axis reports the different rapid tests challenged. The horizontal axis reflects the odds ratio magnitude.

The same scheme to compare the tests under the manufacturers' protocol was then applied to compare their performance under the water protocol. A simple logistic model was fitted for each test. All seven rapid tests performed better under the manufacturers' dilution protocol than under the 50% w/v protocol (Figure 2), as already suggested by the descriptive analysis, however, upon the least approach, this result appeared to be statistically significant only for the *AJ Roboscreen® BetaPrion* and the *Bio-Rad® TeSeE™ SAP* kits.

Assessment of test specificity was not within the scope of this study; nevertheless, appropriate BSE-negative tissue amounts tested on each test platform displayed negative results.

## Discussion

In this study we have evaluated the analytical sensitivity of approved rapid tests for the current known atypical BSEs detection. It is to be noted that Seuberlich et al. [38] raised the possibility that a new prion disease not previously encountered and distinct from the known types of BSEs exists. Nevertheless, the information is really limited and the puzzle of the different observations has still to be assembled, considering that the results described remind the features of poorly digested normal PrP (known as the physiologically C2 fragment of PrP [39], [40]).

Referring to the tissues origin, is to be remarked that the investigated H-BSE tissues originated from intracranially challenged cattle, whereas the three other forms derived from field cases. Nevertheless, recent studies showed that biochemical and histopathological features of experimental H-type BSE animals were identical to that found with field H-type [12], [24], [41].

According to our results, all tests were able to detect both H- and L-BSE types at a 1∶16 dilution prepared as directed by the manufacturer's instructions, with the same performance as for classical BSE.

The LOD varied across the tests. The *IDEXX® HerdCheck BSE-scrapie short protocol* showed the highest analytical sensitivity, as previously reported in a EURL study on classical BSE [21]. The performance of the *AJ Roboscreen® BetaPrion*, *IDEXX® HerdCheck BSE-scrapie ultra short protocol*, and *Prionics® - Check WESTERN* compared favourably with one another at our statistical analysis. The *Prionics® - Check PrioSTRIP*, *Bio-Rad® TeSeE™ SAP* and *Roche® PrionScreen tests* showed the lowest sensitivities for all the BSE types analyzed. These results were confirmed also using other explorative statistical approaches (e.g., Poisson models for number of positive replicates, receiver operating characteristic [ROC] curves) which we had initially applied (results not reported).

The analytical sensitivity of the tests was investigated in accordance with the requirements set by the relevant evaluation protocols established by the European Commission, the SSC and EFSA, using serial dilutions of sample replicates.

Test differences between the last positive dilutions of weak and strong C-BSE samples varies among the different systems from two to four factors (2 base logarithm) for buffer dilutions and from two to five factors for water dilutions. In this context, the different tests showed parallel results between the dilutions prepared following the two protocols. The dynamic range of each rapid test or rather the concentration range of PrP^res^ that results in a change in response is a specific peculiarity of each diagnostic system.

The rate of conversion of substrate to coloured product should be proportional to the amount of PrP^res^ within the well, but there are many limits to this depending on the analyte itself, that tends to aggregate rapidly in solution, and on the combination of methods and materials used within the test kits other than on the equipments.

A gradual stratification of the signal represents a *surplus* value for TSE rapid assays.

In our study, the *Bio-Rad® TeSeE™ SAP* test could surprisingly detect only the 1∶2 dilution when challenged with positive C BSE weak samples. A loss of analytical sensitivity for this test was observed also during the active surveillance activity carried out from 2004 to 2008 by the Italian Reference Center for TSEs applying *Bio-Rad® TeSeE™* test. In that context, a National batch testing was performed on every new batch prior to commercialization to provide reassurance that BSE rapid test kits were fit for the survey purpose. As a consequence, distribution of some kit batches was precluded because of the lack of signal showed on positive reference samples. Further to the unexpected poor performance of *Bio-Rad® TeSeE™* within this study even after test repetition, the same *Bio-Rad®* homogenate sample set, according to previous studies in which its suitability for the *IDEXX* test was shown, was challenged with the last test revealing signals miming the ones reported *for IDEXX* test (data not shown). The question of whether the specific kit batch affected the test performance is of concern, but it is noteworthy that all producers were asked to provide a kit for this evaluation. Thereby, our results represent a picture of the kits available on the market.

The seven simple logistic models showed a meaningful difference between the dilution protocols only for the *AJ Roboscreen® BetaPrion* and *Bio-Rad® TeSeE™ SAP*. The lower bounds of the 95% confidence intervals for the *Roche® PrionScreen* and *Prionics® - Check WESTERN* tests approached 1 (0.9528 and 0.9755, respectively); for the remaining tests, there was no statistical evidence of a higher test sensitivity between the manufacturer's dilution protocol and the 50% w/v protocol (Figure 2).

Whenever in order to evaluate the field performances of BSE rapid *post mortem* tests the manufacturers' protocol represents the term of reference, the relevance of water dilution-based results relies on the specific Annex X of Regulation (EC) 999/2001 requirements. NRLs for TSE periodically have to verify national diagnostic standards and methods by means of comparative trials. The objectives are to monitor national rapid test activity and to demonstrate to the EC that the rapid surveillance system is effective. EURL itself annually verifies the interlaboratory agreement of the rapid systems used by the NRLs.

As previously reported in the EURL study [20], the analytical sensitivity values obtained under the 50% w/v protocol were from one to three dilutions inferior to those obtained under the specific homogenization protocol. For all the tests except one, the discrepancies between the two modes of dilutions were similar whatever the sample tested. Particularly with the Bio-Rad test the strong positive C -BSE sample was four factors lower when the water protocol was applied. Anyway, this is congruent with the EFSA 2009 results [21], where the discrepancy set at three logarithms. This difference needs to be taken into account when organizing ring trials, during which a less sensitive test could be penalized.

To rule out a possible decrement of the signal related to the storage of the water aliquots, and because of the scarcity of atypical BSE material, the laboratory test exercise was completed within a 15-day period. This precautionary approach was taken as no data exist on the stability of atypical BSE homogenates, whereas differences in stability have been observed for atypical *versus* classical scrapie [21], [42], [43], [44]. Further, as it is indeed known that the results of some tests can lapse while approaching the expiring date of kit batches, the kits provided for the evaluation were expected to expire from three to six months after the date of testing. Table S1 lists the kit batches used, the expiring dates and the days of testing.

With regard to the homogeneity of serial dilutions, as PrP^res^ is amyloidogenic, the fibrils tend to aggregate in solution [45], thus potentially hindering a real homogeneity of dilution series. In our study, the ICC of the replicates was higher than 0.99. This ensured that, whenever the amounts of BSE tissues available were extremely limited, the material tested was homogeneous.

When considering the working principle of rapid tests, summarized in the Text S1, all approved tests include a PK digestion step to unmask cryptic epitopes, except for the *IDEXX HerdChek® BSE-scrapie EIA*, which relies on conformational detection technology using a specific aggregate specific capture ligand on a dextran polymer (Seprion ligand technology, Microsens Biotechnologies, London, UK) [46]. The severe effects of proteinase K (PK) in digesting atypical PrP^res^ are well known. Depending on the PK concentration, signal loss after atypical BSE-related PrP^res^ PK digestion varies from less than 20% for the C-type isolates to more than 50% for both L- and H-type BSE tissues [4]. This could be the reason for the higher sensitivity of the IDEXX test in detecting atypical BSEs compared to the others. However, the type of detergent used in homogenates and the type of TSE strain used do affect the extent of PrP^res^ degradation, and this remains a matter of further study [47].

With regard to the interpretation of results, five of the rapid tests in this study are based on semi-quantitative ELISA methods that produce a qualitative result relative to a cut-off value. To minimize subjectivity, the study's *Prionics® - Check PrioSTRIP* results were interpreted with the use of the computerized *PrioSCAN*® software, although visual interpretation by two independent readers was also validated. The *Prionics® - Check Western* is both a qualitative and quantitative test, as it distinguishes PrP^res^ in non-, mono-, and diglycoforms while expressing their respective quantitative ratio and migration positions. The diagnostic criteria for positive results are based on the exhibition of a three-band signal, the top one corresponding to a protein with an approximate molecular weight of 30 kD. Signal intensity decreases from top to bottom, but the higher band should be clearly visible immediately under the PK band. Significant blot images of atypical BSE dilution series obtained on in the frame of this study are presented in the Figure S1. In addition, extremely weak samples, notably for atypical BSE strains, can vary in their conventional blot pattern that fit positive criteria. Glycoform separation on the *Sodium Dodecyl Sulphate PolyAcrylamide* gel by electrophoresis causes the PrP^res^ signal to thin out along the migration line rather than concentrate in a narrow area, as occurs with ELISA and immunochromatographic methods. This means that if the relative non-, mono-, and diglycoform immunoreactivity ratios of L-BSE are taken as corresponding roughly to 39%, 35%, and 26% [48], the blot signal characterizing the last tissue ratio meeting the non-negative criteria generates from only 39% of the total prion protein on the migration line. Despite this, the *Prionics® - Check Western* was found to be among the more sensitive systems, indicating that the interpretation of a specific PrP^res^ marker by an expert reader can increase the test's sensitivity.

In conclusion, despite the evidence of clear differences in relative analytical sensitivity, the LOD of all seven rapid tests included in this study, against all the classes of material used, was within a 2 log_10_ range of the best-performing test, thus meeting EFSA criteria for rapid tests for BSE monitoring.

No certain conclusions on the field of diagnostic performance of these rapid-test kits can be drawn from our results on their analytical sensitivity, as the two parameters are not directly linked, anyway samples from animals exhibiting subclinical signs [24], could be expected to behave similarly to extremely diluted CNS tissues used in analytical sensitivity studies.

The outcome of this study endorses the current epidemiological follow up and interpretation of all three BSE forms prevalence [49], [50] and means that for epidemiological studies the data obtained in the different countries and regions of EU can be considered equally, as plausibly, most stronger atypical cases have been detected by the different rapid tests.

## Supporting Information

Figure S1 **Prionics** ***® - Check WESTERN*** **Rapid Test results.**
(DOC)

Table S1 **Kit batches, expiring dates and days of testing.**
(DOC)

Text S1 **Brief description of the principles of the different rapid tests.**
(DOC)
