# Supplementary material for: EU-Approved Rapid Tests for Bovine Spongiform Encephalopathy Detect Atypical Forms: A Study for Their Sensitivities
Source: PLoS One. 2012 Sep 11;7(9):e43133. doi: 10.1371/journal.pone.0043133 (PMC3439472; doi:10.1371/journal.pone.0043133)
Supplement: Figure S1 — Prionics ® - Check WESTERN Rapid Test results. (DOC) [file pone.0043133.s001.doc]

**Figure S1:** Prionics*® - Check WESTERN* Rapid Test results

Serial dilutions of L-BSE and H-BSE tissues from 1:8 to 1:1024 are presented. Due to the test format, dilutions 1:2 and 1:4 were positioned in a separate *PolyAcrylamide* gel (data not shown).

**L – BSE**

**Water dilution series**


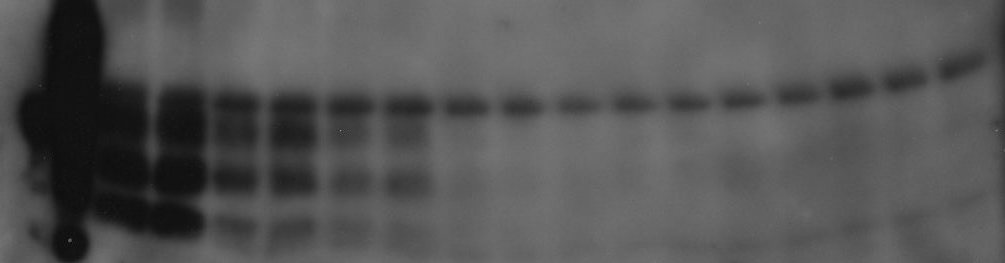


C+ 8 16 32 64 128 256 512 1024

**Manufacturer dilution series**


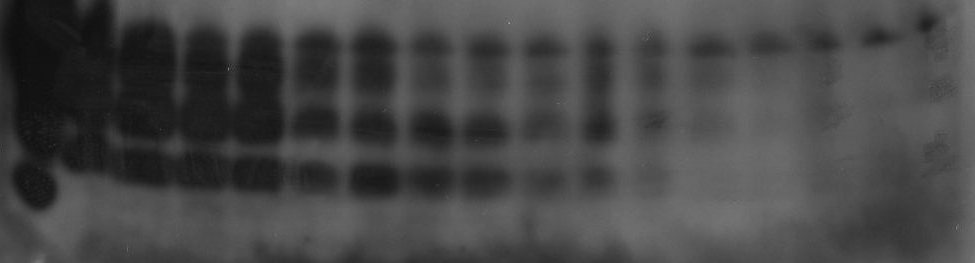


C+ 8 16 32 64 128 256 512 1024

**H – BSE**

**Water dilution series**


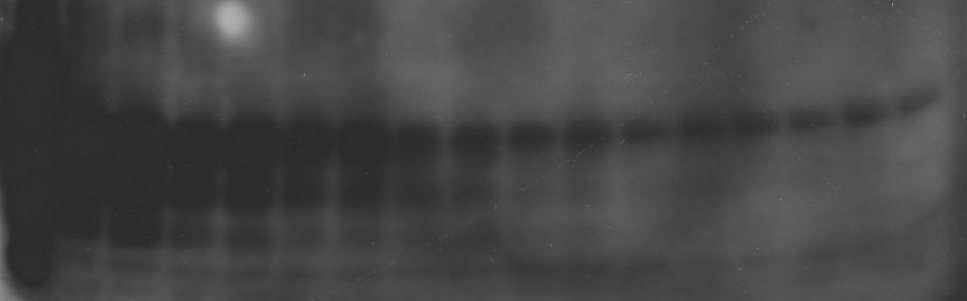


C+ 8 16 32 64 128 256 512 1024

**Manufacturer dilution series**


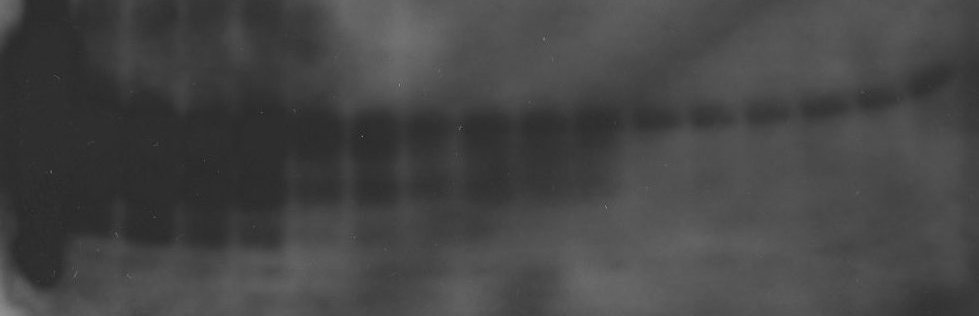


C+ 8 16 32 64 128 256 512 1024
