# Supplementary material for: EU-Approved Rapid Tests for Bovine Spongiform Encephalopathy Detect Atypical Forms: A Study for Their Sensitivities
Source: PLoS One. 2012 Sep 11;7(9):e43133. doi: 10.1371/journal.pone.0043133 (PMC3439472; doi:10.1371/journal.pone.0043133)
Supplement: Table S1 — Kit batches, expiring dates and days of testing. (DOC) [file pone.0043133.s002.doc]

| **Test Batch Expiry date Date of testing** |
| --- |
| Prionics –Check Priostrip S100201A 11 Feb. 2012 31 Mar. 2011 |
| BetaPrion BSE EIA Test Kit 1110-01 30 Nov. 2011 25 Mar. 2011 |
| Prionics –Check Western W101101B 30 Nov. 2011 21 Mar. 2011  22 Mar. 2011  23 Mar. 2011  24 Mar. 2011  25 Mar. 2011 |
| TeSeE BioRad Purification 1A0083 15 Dec. 2011 04 Apr. 2011  Detection 1A1037 |
| Idexx HerdChek BSE HF614 27 Aug. 2011 28 Mar. 2011  04 Apr. 2011 |
| Roche PrionScreen 12475600 29 Feb. 2012 30 Mar.2011 |

**Table S1:** Kit batches, expiring dates and days of testing
