# Supplementary material for: EU-Approved Rapid Tests for Bovine Spongiform Encephalopathy Detect Atypical Forms: A Study for Their Sensitivities
Source: PLoS One. 2012 Sep 11;7(9):e43133. doi: 10.1371/journal.pone.0043133 (PMC3439472; doi:10.1371/journal.pone.0043133)
Supplement: Text S1 — Brief description of the principles of the different rapid tests. (DOC) [file pone.0043133.s003.doc]

**Text S1** Brief description of the principles of the different rapid tests

**Prionics*® - Check WESTERN***

Is an immuno-blotting test based on a Western blotting procedure for the detection of the proteinase K-resistant fragment PrP^res^. The Prionics®-Check WESTERN achieves its result by monitoring three independent criteria: protease-resistance, glycosylation pattern and lower molecular weight of the protease-resistant PrP^res^-fragment (27-30 kD) compared to normal, undigested PrP. After sample collection the test follows a five step protocol, consisting of homogenization, protease digestion, gel electrophoresis, blotting and immunological detection.

***Bio-Rad® TeSeE^TM^ SAP***

Is a sandwich immunoassay for PrP^res^ detection (short assay protocol) carried out following denaturation and concentration steps. A purification and a detection kit are provided. The Purification Kit allow purification, concentration and solubilisation of PrP^res^. Processing of the samples comprises grinding of samples, treatment with proteinase K, concentration of PrP^res^ by precipitation, solubilisation of PrP^res^ for immunoenzymatic assay using the reagents of the TeSeE™ SAP Detection Kit.

***IDEXX ®HerdChek BSE-scrapie Antigen Test Kit, EIA***

Is an immunoassay using a chemical polymer for selective PrP^res^ capture and a monoclonal detection antibody directed against conserved regions of the PrP molecule. The kit uses a proprietary method that allows detection of abnormal prions. A PrP^res^-specific ligand is immobilized on the surface of the BSE antigen-capture plate. Test samples are prepared by homogenizing the tissues and then diluting the sample with working plate diluent. After the sample is applied to the plate, the disease-associated conformer binds to the immobilized ligand with high affinity. Colour development is related to the relative amounts of PrP^res^ captured by the ligand immobilized in the microtiter plate well.

***Prionics® - Check PrioSTRIP***

Is a lateral-flow immunochromatographic immunoassay using two different monoclonal antibodies to detect proteinase K-resistant PrP fractions. The test follows a four step protocol, consisting of homogenization, protease digestion, pre-incubation and detection. Results can be interpreted with the use of the computerized *PrioSCAN*® software, although a visual interpretation by two independent readers is also validated.

***AJ Roboscreen® BetaPrion***

Is a two-sided immunoassay using two different monoclonal antibodies directed against two epitopes presented in a highly unfolded state of bovine PrP Sc. Specimens of bovine brain are homogenized and incubated with proteinase K. Solubilzed PrP^res^ is captured by a specific monoclonal anti-PrP antibody coated to the wells of a microtitre strip. Bound PrP^res^ is detected with a HRP-conjugated anti-PrP antibody. The wells are washed and a substrate solution is added. The developed colour indicates the existence of PrP^res^ in the specimen in comparison to a negative and a positive control in case of overshoot the declared.

***Roche® PrionScreen***

Is a sandwich ELISA for the detection of proteinase K-resistant PrP^res^. The test uses Proteinase K for the digestion and removal of the test-interfering common form of Prion Protein and an Enzyme-Linked ImmunoSorbent Assay (ELISA) for the detection of the remaining PrP^res^ in streptavidin-coated microplates.
